# Convergent and criterion validity of PROMIS anxiety measures relative to six legacy measures and a structured diagnostic interview for anxiety in cancer patients

**DOI:** 10.1186/s41687-022-00477-4

**Published:** 2022-07-20

**Authors:** Kerrie Clover, Sylvie D. Lambert, Christopher Oldmeadow, Benjamin Britton, Alex J. Mitchell, Gregory Carter, Madeleine T. King

**Affiliations:** 1 Psycho-Oncology Service, Calvary Mater Newcastle, Waratah, NSW Australia; 2 College of Health, Medicine and Wellbeing, School of Medicine and Population Health, University of Newcastle, Callaghan, NSW Australia; 3 Ingram School of Nursing, McGill University & St. Marys’ Research Centre, Montreal, QC Canada; 4 Clinical Research Design, Information Technology and Statistical Support (CReDITSS) Hunter Medical Research Institute, New Lambton Heights, NSW Australia; 5 Cancer & Molecular Medicine, University of Leicester, Leicester, UK; 6 Faculty of Science, School of Psychology, University of Sydney, Level 6 North, Chris O’Brien Lifehouse (C39Z), Camperdown, NSW 2006 Australia

**Keywords:** Cancer, Anxiety, Validation study, Anxiety disorders, Psycho-oncology, Psychology, Patient-reported outcome measures, Reproducibility of results, Surveys and questionnaires

## Abstract

**Background:**

Detecting anxiety in oncology patients is important, requiring valid yet brief measures. One increasingly popular approach is the Patient Reported Outcomes Measurement Information System (PROMIS); however, its validity is not well established in oncology. We assessed the convergent and criterion validity of PROMIS anxiety measures in an oncology sample.

**Methods:**

132 oncology/haematology outpatients completed the PROMIS Anxiety Computer Adaptive Test (PROMIS-A-CAT) and the 7 item (original) PROMIS Anxiety Short Form (PROMIS-A-SF) along with six well-established measures: Hospital Anxiety and Depression Scale-Anxiety (HADS-A); Generalised Anxiety Disorder-7 (GAD-7); Depression, Anxiety and Stress Scale-Anxiety (DASS-A) and Stress (DASS-S); Distress Thermometer (DT) and PSYCH-6. Correlations, area under the curve (AUC) and diagnostic accuracy statistics were calculated with Structured Clinical Interview as the reference standard.

**Results:**

Both PROMIS measures correlated with all legacy measures at *p* < .001 (Rho = .56–.83). AUCs (> .80) were good for both PROMIS measures and comparable to or better than all legacy measures. At the recommended mild cut-point (55), PROMIS-A-SF had sensitivity (.67) comparable to or better than all the legacy measures, whereas PROMIS-A-CAT sensitivity (.59) was lower than GAD-7 (.67) and HADS-A (.62), but comparable to PSYCH-6 and higher than DASS-A, DASS-S and DT. Sensitivity for both was .79. A reduced cut-point of 51 on both PROMIS measures improved sensitivity (.83–.84) although specificity was only adequate (.61–.62).

**Conclusions:**

The convergent and criterion validity of the PROMIS anxiety measures in cancer populations was confirmed as equivalent, but not superior to, established measures (GAD-7 and HADS-A). The PROMIS-A-CAT did not demonstrate clear advantages over PROMIS-A-SF.

## Plain English summary

Many people with cancer suffer needlessly from intense anxiety, since anxiety can be treated. However, in order to be treated, anxiety has to be recognised. Asking people to fill-in a questionnaire about their feelings is one way to let their health care team know if they feel overly anxious. This can lead to a conversation about their feelings, support, and treatment.

The question for this study is – which questionnaire should we use? Many questionnaires try to measure anxiety, but none is perfectly accurate. A group of North American researchers (the PROMIS group), have used advanced techniques as a new way of developing questionnaires. We tested whether these new, PROMIS anxiety questionnaires were accurate for people with cancer.

We used a gold standard comparison—an interview by a psychologist. We also compared the new questionnaires to six well-established questionnaires. We found the PROMIS anxiety questionnaires were accurate, but that they were not better (more accurate or shorter) than two existing measures. We also found that the mathematical models used to score the PROMIS anxiety questionnaires might need adjusting for people with cancer.

## Background

Addressing psychosocial concerns is a well-established standard of care in cancer [1]. Anxiety disorders are common among people with cancer, with prevalence estimates ranging from 18 to 30% [2–6]. Untreated anxiety disorders can have multiple clinical consequences and effective treatments are available [7].

To treat anxiety disorders, they must first be detected [8, 9]. Several validated patient-reported outcome measures (PROMs) for anxiety have been used in oncology; however, none has demonstrated perfect accuracy, leaving scope to develop new measures. One comprehensive approach to developing new PROMs is the PROMIS initiative (8), which applied item-response theory to create patient-reported outcome measures. For anxiety, two types of PROMIS measures are available: “short form” (PROMIS-A-SF) measures in the traditional format and a measure using computer adaptive technology (CAT) (PROMIS-A-CAT). There are several short-form options (2, 4, 6 and 8 items) along with an original, seven-item version and users can also create their own short forms [10]. Longer measures achieve higher correlations with the full item bank, but all versions have highly similar precision and reliability [10]. CAT offers the benefit of tailoring item presentation depending on the patient’s prior responses, reducing response burden by enabling the smallest possible number of items to be used to achieve a specified degree of precision [11].

PROMIS measures are increasingly used in oncology [12], with a recent systematic review identifying 31 studies that used the PROMIS anxiety measures as PROs [12]. However, the concurrent and criterion validity of the PROMIS anxiety measures has not been fully established in oncology populations. A few studies have reported concurrent validity against anxiety-specific ‘legacy’ PROMs, that is, validated measures that preceded PROMIS. We found two studies that used the PROMIS-A-CAT in oncology [13, 14]; however, neither included legacy measures, or reported on CAT functioning.

Three studies have supported the convergent validity of PROMIS-A-SF measures, by finding moderate correlations with mental-health-related legacy measures in oncology samples [15–17]. Quach [15] reported moderate correlations between a five-item version of the PROMIS-A-SF and a prostate-cancer specific anxiety questionnaire (.44) and a general mental health summary questionnaire (.59). Wilford [16] reported moderate correlations (.45–.66) between the PROMIS-A-SF (7 item) and three of four general measures of mental health (FACT emotional wellbeing, Impact of Events and Perceived Stress) and a strong (.72) correlation with the remaining instrument (Brief Symptom Inventory). Sikorskii [17] also reported a moderate correlation (approximately 0.60) between the State Anxiety questionnaire and a four-item PROMIS-A-SF measure.

In terms of criterion validity, five studies examined the diagnostic accuracy of PROMIS anxiety measures, using structured clinical interviews as the reference standard [18–22]; however, none were in oncology. Four used the PROMIS-A-SF (8-item version) [18–21] and one used the full item bank (29 items) [22]. No study used the PROMIS-A-CAT. Criterion validity of the PROMIS-A-SF was supported [18–21] with “acceptable” areas under the curve (AUCs) (.85–.86). Similar AUCs were obtained for the GAD-7 and HADS-A, indicating that PROMIS-A-SF had similar performance to these legacy measures. Recommended optimal cut-points on the PROMIS-A-SF varied, from 55 to 60.

As none of these studies involved an oncology sample and none used the PROMIS-A-CAT, further validation of the PROMIS anxiety SF and CAT measures in oncology populations is warranted.

### Aims

Our primary aim was to examine the criterion validity of the PROMIS-A-SF and PROMIS-A-CAT among oncology outpatients by comparison with structured interview diagnosis of Any Anxiety Disorder. Our second aim was to assess the convergent validity of the PROMIS anxiety measures relative to six legacy measures of anxiety and emotional distress. Our third aim was to describe the functioning of the PROMIS-A-CAT.

## Methods

### Patients and setting

A convenience sample of 132 oncology outpatients was recruited from a regional Australian cancer centre. All patients attending for cancer care were eligible, with the following exclusion criteria: first clinic visit; insufficient English language skills; too unwell to consider participation, in the opinion of the clinic nurse. Ethics Committee approval was obtained (Reference 09/11/18/5.09). Depression measures were also administered, to examine the criterion and convergent validity of PROMIS depression measures, which have previously been reported [23].

### Procedure

Patients were approached in the clinic waiting room. Consenting participants returned to complete a structured diagnostic interview conducted by a Registered Psychologist, who then introduced a different staff member to independently facilitate patient completion of the legacy measures, using the QUICATOUCH platform [24], followed by PROMIS measures on the Assessment Centre platform [25].

### Measures

Two PROMIS anxiety measures (PROMIS-A-CAT and PROMIS-A-SF) and six legacy measures were administered to all participants. Legacy measures were three measures of anxiety (GAD-7, HADS-A, DASS-A); one stress measure (DASS-S); and two emotional distress measures (Distress Thermometer, PSYCH-6), and were presented in fixed order (DT, GAD-7, PSYCH-6, HADS, DASS). Higher scores indicated more severe symptoms on all measures.

#### PROMIS measures

Both PROMIS measures record the frequency of symptoms over the past seven days on a five-point response scale (0–4).

##### PROMIS Anxiety CAT (PROMIS-A-CAT)

The PROMIS-A-CAT selects items from a 29-item bank [25], focussing on fear, worry, dread, hyperarousal (tension, restlessness) and somatic arousal (cardiovascular symptoms, dizziness). The PROMIS default settings for standard error (0.3) and maximum number of items (12) were used and the minimum number of responses was changed to one item (from the default of four) to allow the lowest possible number of items to be asked. The Assessment Centre website was used to administer the CAT (using the full 29-item bank) and transform the raw, summed scale scores into T-Scores using norms (mean = 50, standard deviation (SD) = 10) [11].

##### PROMIS Anxiety Short Form (PROMIS-A-SF)

We used version 1 of the PROMIS-A-SF, a seven-item subset of the PROMIS-CAT 29-item bank. Item scores were summed to obtain the total raw score which was then converted to a T-score (mean 50, SD = 10) [26] and rounded to the nearest digit for ease of reporting. A minimum of six completed items was required and in this case, the total score was imputed following instructions in the PROMIS scoring guide [27] (http://nihpromis.org/measures, accessed Nov 2013).

To examine diagnostic accuracy, T-scores from both measures (PROMIS-A-CAT and PROMIS-A-SF) were categorised into severity level using cut-points recommended for the PROMIS total item bank [28] as follows: normal 0–54; mild 55–64; moderate 65–74 and 75 + severely symptomatic.

#### Legacy anxiety and distress measures

The GAD-7 was developed to measure Generalised Anxiety Disorder (GAD) symptoms [29] and also has established diagnostic accuracy for panic disorder, social anxiety and PTSD [30]. It is a seven-item scale, with each item rated from zero (none of the days) to three (nearly every day) over the past two weeks, giving a response range of 0–21 (PHQ Instruction Manual https://www.phqscreeners.com/images/sites/g/files/g10016261/f/201412/instructions.pdf). Recommended cut-points are 5–9 (mild), 10–14 (moderate) and 15 or more (severe). A score of 10 or more is considered an appropriate cut-point for screening for anxiety disorders. Cronbach’s alpha for the GAD in this sample was 0.93.

The Hospital Anxiety and Depression Scale (HADS) is a 14 item measure, including a seven item anxiety subscale (HADS-A), that has been widely used in oncology [31–33]. Items are rated by how often they occurred over the past two weeks, with zero indicating less of the time and three indicating more of the time (response range 0–21). Possible anxiety is indicated by a score of 8–10, with a score of 11 or more indicating probable anxiety [33]. Cronbach’s alpha was 0.88.

The anxiety (DASS-A) and stress (DASS-S) subscales of the Depression Anxiety and Stress Scale (DASS) are seven-item sub-scales of the 21 item DASS scale. Each item is rated on a four-point scale, from 0, (did not apply to me at all) to 3 (applied to me very much, or most of the time) over the past week [34]. The sub-scale score is doubled for categorisation (range 0–42). Anxiety symptoms are categorised as mild (8–9); moderate (10–14); severe (15–19) and extremely severe (20 and over); stress symptoms are categorised as mild (15–18); moderate (19–25); severe (26–33) and extremely severe (34 and over). Cronbach’s alpha for DASS-A was 0.78 and for DASS-S was 0.90.

The PSYCH-6 is the six-item psychological symptom subscale of the Somatic Psychological Health Report (SPHERE) [35]. Respondents indicate how often items have troubled them over the past few weeks from zero (some of the time or never) to two, (most of the time), (response range 0–12). We have previously established a cut-point score of three or more for oncology outpatients [35]. Cronbach’s alpha was 0.93. The Distress Thermometer (DT) is a one-item rating of distress over the past week on a 0–10 scale with an established cut-point score of four or more [36].

#### Structured diagnostic interview

The reference standard was the Anxiety Disorders module of the Structured Clinical Interview for DSM-IV-TR Axis I Disorders (SCID) to assess whether respondents met criteria (present vs absent or sub-threshold) in the past month for Any Anxiety Disorder: Panic Disorder; Agoraphobia; Social Phobia; Specific Phobia; Obsessive–Compulsive Disorder; Posttraumatic Stress Disorder or Generalized Anxiety Disorder.

The interview was conducted by one of two trained, Registered Psychologists with experience in adult mental health. Training followed SCID recommendations, with private study, videotape training, role-play and inter-rater comparisons of a video tape to ensure proficiency [37]. Training was supervised by a Clinical Psychologist (BB) with extensive experience in psycho-oncology. The SCID interviewers were blind to participants’ responses on the self-report measures.

### Statistical analysis

Analyses used Stata V14 (Statacorp, College Station, TX) and Statistical Package for the Social Sciences 22.0.

#### Criterion Validity

##### Area Under the Curve (AUC)

To address our primary aim, we conducted ROC analysis using a SCID diagnosis of Any Anxiety Disorder in the past month as the reference standard, calculating AUC to determine each measure’s performance. AUC were classified as: > 0.70 = ‘useful’; > 0.80 = ‘good’ and > 0.90 = ‘very good’ [38]. Results for each legacy measure at recommended cut-points were calculated to provide a context for interpreting the performance of the PROMIS measures.

##### Measures of diagnostic accuracy

For the PROMIS-A-CAT and PROMIS-A-SF, we calculated sensitivity, specificity, negative predictive value (NPV) and positive predictive (PPV) at the T score cut-points for mild (55) and moderate (65) anxiety proposed by Cella et al. [28]. Too few people were classified with severe anxiety to warrant analysis. To examine cut-points we used Youden’s Index, a commonly used summary measure of sensitivity and specificity [39] ranging from zero to one, where one represents perfect performance. Youden’s index assumes equal importance of sensitivity and specificity and therefore may not be optimal for anxiety screening programs [40], which might give higher weight to sensitivity, so that cases are not missed. Nevertheless, we used it to allow comparison with previous studies [18–21].

#### Convergent validity

To address our second aim, two series of bivariate (Spearman) correlations were calculated, comparing the legacy measures with the PROMIS-A-CAT T-Score and PROMIS-A-SF T-Score, respectively. Correlations were classified as weak (< .4), moderate (.4 to < .7) and strong (.7–1) (41). Spearman’s correlation was chosen as data were non-normal.

#### PROMIS-A-CAT functioning

Our final aim was addressed with descriptive statistics: mean and range of PROMIS-A-CAT scores by SCID diagnosis group (Any Anxiety Disorder vs none); mean and range of number of items administered by the PROMIS-A-CAT for the total sample, by SCID diagnosis (Any Anxiety Disorder vs none) and by PROMIS-A-CAT anxiety severity categories (28).

## Results

### Sample

Of 322 eligible people indicating initial interest, 168 attended for data collection, 139 of these commenced the PROMIS measures (PROMIS measures were added some weeks after initial recruitment began) and 132 contributed to this analysis with data from the SCID Anxiety module plus at least one PROMIS measure and at least one legacy measure. The SCID interview took a median of 28 min (IQR 20-35); median time to complete legacy measures was 29 min (IQR 18-53) and 13 min (IQR 11-17) for PROMIS measures.

The sample was predominantly female (69%) and married (68%) (Table 1). Time since diagnosis ranged from five weeks to 21 years (median = 74 weeks).

**Table 1:** Sample characteristics (n = 132)

Variable	n	%
Female	91	69
Marital status		
Married/living as married	90	68
Separated/divorced/widowed/never married	42	32
Time since diagnosis		
0–12 months	49	37
13–24 months	30	23
More than 24 months	53	40
Cancer stage		
Early	19	14
Stage 2 or 3	30	23
Advanced	23	15
Could not determine from self-report	63	48
Cancer type		
Breast	59	45
Haematological	18	13
Colorectal	16	12
Lung	13	10
Other	26	20
SCID diagnosis		
Generalised anxiety disorder	15	11
Specific phobia	13	10
Social phobia	11	8
Post-traumatic stress disorder	5	4
Agoraphobia without panic	3	2
Panic disorder	2	2
Obsessive compulsive disorder	2	2
Any anxiety disorder	37	28

#### SCID diagnosis

Thirty seven (28%) participants met criteria for Any Anxiety Disorder in the past month on the SCID (Table 1). The most common anxiety diagnoses were GAD (11%); specific phobia (10%) and social phobia (8%). Ten (8%) participants met criteria for more than one anxiety disorder.

### Criterion validity

#### AUC

AUC were in the ‘good’ range for the PROMIS-A-CAT (0.82, 95%CI = 0.73-0.90) and PROMIS-A-SF (0.80, 95%CI = 0.71-0.89) (Table 2). The AUC for the GAD-7 (0.84) was also in the good range while the AUC for all other legacy measures fell into the ‘useful’ range (0.72 to 0.79).

**Table 2:** Area under the curve (AUC) for each measure versus diagnosis of any anxiety disorder using the structured clinical interview for DSM disorders (SCID)

Measure	AUC (95% CI)
PROMIS-A-CAT T score	.82 (.73–.90)
PROMIS-A-SF T score	.80 (.71–.89)
GAD-7	.84 (.77–.91)
HADS-A	.79 (.70–.89)
PSYCH-6	.79 (.70–.89)
DASS-S	.77 (.68–.86)
DASS-A	.72 (.62–.82)
DT	.72 (.62–.82)

n = 122 with all measures

#### Diagnostic accuracy statistics

**Fig. 1:**
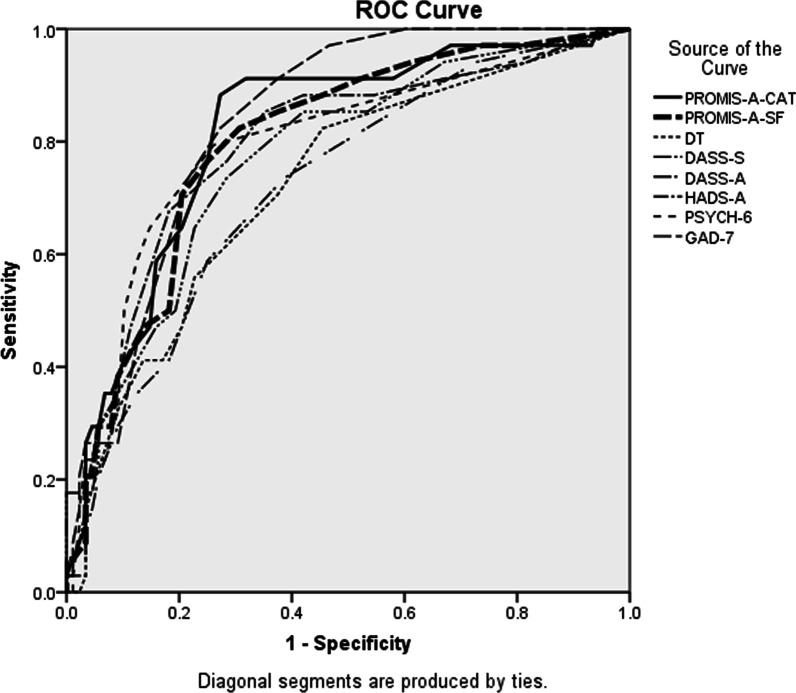
Area under the curve (ROC) curves of all patient-reported measures versus a SCID diagnosis of Any Anxiety Disorder

ROC curves for all measures (Fig. 1) provide context for interpreting PROMIS measures’ performance. The mild (55) cut-point on the PROMIS-A-CAT had sensitivity of 0.59 and specificity of 0.79 (Table 3) and the PROMIS-A-SF had higher sensitivity (0.67) and identical specificity (0.79). At the moderate cut-point (65) both PROMIS measures had sensitivity of 0.19 and specificity of 0.97.

**Table 3:** Diagnostic accuracy of legacy measures at established cut-points compared with diagnosis of Any Anxiety Disorder using the Structured Clinical Interview for DSM disorders (SCID)

Measure	Sensitivity	(95% CI)	Specificity	(95% CI)	PPV*	(95% CI)	NPV**	(95% CI)
Mild cut-points								
PROMIS-A-CAT 55	.59	(.43–.76)	.79	(.70–.86)	.53	(.41–.65)	.83	.78–.89
PROMIS-A-SF 55	.67	(.50–.81)	.79	(.71–.87)	.56	(.44–.68)	.86	.80–.92
HADS-A possible	.62	(.47–.78)	.80	(.72–.88)	.55	(.40–.70)	.84	.77–.92
DASS-A mild	.39	(.23–.55)	.78	(.69–.86)	.40	(.24–.56)	.77	.68–.85
DASS-S mild	.27	(.13–41)	.94	(.88–.99)	.63	(.39–.86)	.76	.68–.84
GAD-7 mild	.68	(.54–.83)	.75	(.67–.84)	.52	(.38–.66)	.85	.78–.93
DT	.46	(.30–.62)	.78	(.70–.86)	.45	(.29–.61)	.79	.70–.87
PSYCH-6	.57	(.41–.73)	.85	(.78–.92)	.59	(.42–.75)	.84	.77–.92
Moderate cut-points								
PROMIS-A-CAT 65	.19	(.08–.32)	.97	(.93–1.00)	.71	(.43–1.00)	.75	.73–.79
PROMIS-A-SF 55	.19	(.08–.33)	.97	(.93–1.00)	.71	(.44–1.00)	.75	.73–.79
HADS-A probable	.27	(.13–.41)	.94	(.89–.99)	.63	(.39–.86)	.77	.69–.84
DASS-A moderate	.33	(.18–.49)	.86	(.79–.93)	.48	(.28–.68)	.77	.69–.85
DASS-S moderate	.16	(.04–.28)	.95	(.90–.99)	.55	(.25–.84)	.74	.66–.82
GAD-7 moderate	.24	(.11–.38)	.95	(.90–.99)	.64	(.39–.89)	.76	.68–.83
Optimal Cut-point***								
PROMIS-A-CAT 53	.81	(.68–.92)	.72	(.63–.81)	.54	(.45–.63)	.91	.84–.96
PROMIS-A-SF 53	.78	(.64–.92)	.70	(.61–.78)	.50	(.42–.60)	.89	.83–.95

^*^PPV Positive Predictive Value; **NPV Negative Predictive Value

^***^ Selected by maximising Youden’s index

At the recommended cut-point (55) for mild anxiety symptoms, the sensitivity of the PROMIS-A-CAT (0.59) was lower than HADS-A and GAD-7, similar to PSYCH-6, and higher than DT, DASS-A and DASS-S (Table 3). Sensitivity of the PROMIS-A-SF (0.67) was comparable to GAD-7, slightly higher than HADS-A, and higher than PSYCH-6, DT, DASS-A and DASS-S. The PROMIS-A-SF had the highest NPV (0.86), similar to the GAD-7, slightly higher than HADS-A and PSYCH-6, which were similar to PROMIS-A-CAT and higher than the DT, DASS-A and DASS-S (Table 3). Thus, the ability of the PROMIS measures to detect anxiety at the proposed mild cut-points varied, with the PROMIS-A-SF comparable to the GAD-7 and HADS-A, but better than the other legacy measures and the PROMIS-A-CAT.

At the proposed moderate cut-point, the specificity of both PROMIS measures (0.97) was higher than those legacy measures with a moderate category (HADS-A, GAD-7, DASS-A and DASS-S), as was the PPV (0.71). However, the sensitivity of the PROMIS measures at the proposed moderate cut-point was very low (both 0.19) and lower than all legacy measures except the DASS-S.

#### Alternate cut-points

Youden’s Index was maximised (J = 0.53) at a cut-point of 53 on the PROMIS-A-CAT with sensitivity of 0.81 and specificity of 0.72. For the PROMIS-A-SF, Youden’s Index was maximised (J = 0.47) over three cut-points (52–54). Sensitivity (0.78) and specificity (0.70) were identical for scores of 52 and 53 while a cut-point of 54 on the PROMIS-A-SF had lower sensitivity (0.72) and higher specificity (0.75). Since there was little to recommend one cut-point over another, the full results for a cut-point of 53 are reported in Table 3, to match the CAT.

A sensitivity analysis was conducted, which included participants with subthreshold anxiety in the reference standard. This included an additional four cases of Any Anxiety Disorder. The results of the study were not substantially changed. AUCs of the PROMIS measures were increased by 0.01–0.03. Sensitivity and specificity were improved by 0.01–0.02. PPV showed an improvement of 0.04–0.1, with a concomitant decrease in NPV (0.01–0.05) which reflects the changed prevalence of anxiety in the sample. Youden’s Index increased by 0.01-0.02 with optimum cut-off points being 1–2 points lower.

### Convergent validity

#### Correlation with legacy measures

Statistically significant (*p* < 0.001) correlations were obtained between the PROMIS anxiety measures and all legacy measures (Table 4). Both PROMIS measures displayed strong correlations with the HADS-A, DASS-S and GAD-7. Moderate correlations were observed between the two PROMIS anxiety measures and DASS-A, DT and PSYCH-6.

**Table 4:** Correlations between PROMIS anxiety measures and legacy measures

	PROMIS-A-CAT T Score		PROMIS-A-SF-T score	
	n	Rho*	n	Rho*
HADS-A	131	.835	128	.820
GAD-7	129	.788	126	.756
DASS-S	128	.769	125	.777
PSYCH-6	128	.697	125	.673
DT	131	.632	128	.598
DASS-A	129	.573	126	.556

^*^All significant at p < .001

### PROMIS-A-CAT functioning

T-scores on the PROMIS-A-CAT ranged from 33–75 with mean = 50.8 and standard deviation (SD) = 9.3. Patients with Any Anxiety Disorder had a significantly higher mean T-score on the PROMIS-A-CAT (57.1, SD = 8.3) than those without (48.3, SD = 8.5) (t =  − 5.4, df = 129, *p* < 0.001). Using proposed severity levels for the PROMIS-A-CAT [28], most patients (67.4%, n = 89), scored in the normal (no-anxiety) range, with 25.0% (n = 32) in the mild, 6.8% (n = 9) in the moderate and one participant (0.8%) in the severe range.

The mean number of items answered on the PROMIS-A-CAT was 6.4 (range 3–12). Almost half (45.8%, n = 60) the participants answered six items on the PROMIS-A-CAT. A further quarter (24.4%, n = 32) answered 3–4 items and 14.5% (n = 19) completed the full 12 items (Table 5). Participants with Any Anxiety Disorder answered slightly fewer items (mean = 5.7, SD = 1.6) than those without an anxiety disorder (mean = 6.7, SD = 2.9) (t = 2.1, df = 129, *p* = .04) (Table 5).

**Table 5:** Number of items* answered on the PROMIS Anxiety CAT

	Total Sample			SCID				PROMIS-A-CAT Categories							
			Cumulative	Any Anxiety		No Anxiety		Normal		Mild		Moderate		Severe	
N items	n = 131	%	%	n = 37	%	n = 94	%	n = 89	%	n = 32	n%	n = 9	n%	n = 1	%
3	14	10.7	10.7	4	10.8	10	10.6	3	3.4	9	28.1	2	22.2	0	0
4	18	13.7	24.4	5	13.5	13	13.8	13	14.6	1	3.1	4	44.4	0	0
5	4	3.1	27.5	0	0	4	4.3	4	4.5	0	0	0	0	0	0
6	60	45.8	73.3	24	64.9	36	38.3	38	42.7	19	59.4	2	22.2	1	100
7	11	8.4	81.7	2	5.4	9	9.6	7	7.9	3	9.4	1	11.1	0	0
8	2	1.5	83.2	1	2.7	1	1.1	2	2.2	0	0	0	0	0	0
9	2	1.5	84.7	0	0	2	2.1	2	2.2	0	0	0	0	0	0
10	0	0.0	84.7	0	0	0	0	1	1.1	0	0	0	0	0	0
11	1	.8	85.5	0	0	1	1.1	17	19.1	0	0	0	0	0	0
12	19	14.5	100.0	1	2.7	18	19.1	2	2.2	0	0	0	0	0	0

*Number of items in other anxiety measures: PROMIS-A-SF (8), HADS-A (7 for anxiety subscale), GAD-7 (7), DASS-A (7 for anxiety subscale), DASS-S (7 for stress subscale), DT (1), PSYCH-6 (6)

PROMIS-A-SF T-scores ranged from 36–74 with mean = 50.6 and SD = 9.2. Patients with Any Anxiety Disorder had a significantly higher mean T-score on the PROMIS-A-SF (56.9, SD = 8.1) than those without (48.1, SD = 8.4) (t(126) =  − 5.4, *p* < .001).

## Discussion

This study confirmed the convergent and criterion validity of two PROMIS anxiety measures, PROMIS-A-CAT and PROMIS-A-SF, by comparing them with four legacy measures of anxiety and two legacy measures of general emotional distress, and against the reference standard SCID for Any Anxiety Disorder in an oncology outpatient sample. It also described how the PROMIS-A-CAT performed in an oncology population, in terms of number of items presented and mean T-scores for those with and without a diagnosis of Any Anxiety Disorder.

### Criterion validity

Criterion validity of both PROMIS anxiety measures was confirmed, with good AUC (CAT = 0.82, SF = 0.80), that was comparable to or better than all legacy measures (0.72-0.84). Five similar validation studies [18–22] in other health conditions, also found that the PROMIS-A-SF had good AUC (0.80-0.86), comparable to or better than the GAD-7 and HADS-A.

#### Cut-points

At the recommended cut-point for mild anxiety (55), the sensitivity and NPV of the PROMIS-A-SF was comparable to the best-performing legacy measures (GAD-7 and HADS-A), followed by the PROMIS-A-CAT. Overall, however, sensitivity (< .67) and NPV (< .86) were relatively low. The optimum cut-point, using Youden’s index, was 53 on the PROMIS-A-CAT and 52–54 on the PROMIS-A-SF. Of four prior PROMIS-A-SF validation studies, two identified similar cut-points (54.3 [20] and 55.4 [21]) and two identified higher cut-points of 59.4 [19] and 59.9 [18].

Several factors may underpin the different findings among studies comparing the SCID, PROMIS-A-SF, HADS-A and GAD-7. Firstly, the studies covered a diverse range of medical issues (heart failure, MS, inflammatory bowel disease, rheumatoid arthritis and cancer). Thus, differences in results may reflect genuine differences between these illnesses and the need to adapt cut-points for particular illness groups. Methodological differences may also be important. In particular, reference standards varied somewhat, with some studies using GAD and others using Any Anxiety Disorder. Further, Any Anxiety Disorder included different diagnostic groups across studies. For example, Fischer [18] excluded PTSD and OCD while we included these diagnoses and Marrie [20] included “anxiety due to general medical condition” and “due to substance use” where we did not. Further research is needed to establish optimum cut-points across illness groups, ideally with standardised reference conditions and consideration of specific anxiety diagnoses.

### Relative value of PROMIS instruments

Our results indicate that the PROMIS anxiety measures perform similarly to the GAD-7 and HADS-A, in terms of convergent and criterion validity. However, they do not offer any clear advantages in terms of diagnostic accuracy, response burden or costs over these measures. Additionally, there was no clear advantage of the PROMIS-A-CAT over the 7–item PROMIS-A-SF, either in terms of criterion validity or reduced response burden. Perhaps the lower criterion validity of the PROMIS-A-CAT (compared with the PROMIS-A-SF) was affected by reducing the minimum number of items to one (from four). However, the minimum number of items answered in practice was three and this only occurred for 11% (n = 14) of the sample (Table 5). Although a quarter of respondents answered fewer items on the PROMIS-A-CAT than the PROMIS-A-SF (under our study conditions, which allowed a one item minimum), approximately a quarter answered seven or more items, including 15% who answered as many as 12 items (the most of any instrument in the study). Since the PROMIS-A-SF is simpler to administer, some other benefit would be required to encourage use of the PROMIS-A-CAT in clinical, and even research, settings. This may explain why most of the studies of PROMIS anxiety measures in oncology have used the SF and not the CAT. Originators of the PROMIS instruments have also recently noted that CATs only offer marginally improved efficiency over short-form measures in anxiety [25].

### Convergent validity

Convergent validity was confirmed by strong correlations between PROMIS anxiety measures and two legacy measures of anxiety (HADS and GAD-7). The lower (‘moderate’) correlations with measures of general distress (DT and PSYCH) might be expected since they are intended to measure generalised distress rather than specific anxiety disorder symptoms, and these moderate correlations are therefore adequate. The correlations between PROMIS Anxiety measures and the DT and PSYCH were higher than we previously reported between PROMIS depression measures and those instruments [41], perhaps suggesting that general distress conceptually resembles anxiety more closely than depression.

### Functioning of the CAT

#### T scores

The mean T-scores on the PROMIS-A-CAT (50.8, SD 9.33) matched the reference population norm, as did the PROMIS-A-SF (50.6, SD = 9.2). As 28% of our sample had a clinical diagnosis of Any Anxiety Disorder, we could have expected our mean to be higher. Two other studies that reported CAT T-scores in oncology populations also obtained mean T-scores close to the reference population mean (52.8, SD 9.1) [13] and 47–48 (SD not reported) [14]. The overall mean Anxiety T-score norm was 49.2 (SD 0.2) in one large (n = 5284) American study of people with cancer, using an 11-item SF measure [42]. As no studies have had reference standard measures of clinical anxiety, it is difficult to determine whether anxiety is genuinely comparable to a general population in oncology samples, or whether the algorithm used to calculate T-scores for CAT and SF measures is not optimal for oncology populations.

Respondents with an anxiety disorder had a considerably higher mean T-score than those who did not on both the PROMIS-A-CAT (57, SD = 8.3 vs. 48, SD = 8.5), and the PROMIS-A-SF (57, SD = 8.1 vs. 48, SD = 8.4), confirming the ability of the PROMIS instruments to distinguish between those with anxiety symptoms and those without. Recently published T-Score maps [43] suggest that T-scores around 50 are obtained by those who never experience symptoms and T-scores of 55–60 are obtained by people who experience symptoms rarely or sometimes. Another recent study [44] linked a T-score of 50 to a score of two, on a single item (0–10) rating of anxiety and T-scores of 55–60 to scores of 4–6/10. These studies suggest that a T-score of 50 is indicative of virtually no anxiety symptomatology and a T-score of 55 suggests low level anxiety symptoms. Consequently, it could have been expected that sensitivity of the PROMIS anxiety measures, at a cut-off point of 55, would have been higher than obtained.

Overall, these findings suggest the current T-score algorithm, which uses norms for the US general population, may not be optimal for oncology populations, given that 28% of our sample had a clinical diagnosis of Any Anxiety Disorder yet this was not reflected in the mean T-Score of 50. Furthermore, those without a mental health diagnosis in our study and in that of Quach et al. [15] had a mean score of less than 50. Differences for oncology populations have been considered previously, with the development of a PROMIS anxiety instrument, specific to oncology (PROMIS_Ca_Bank_v1.0-Anxiety 6-24-2016.pdf), however, this instrument does not appear to have been further adopted (Tran 2021).

#### Number of CAT items

A stated benefit of CAT is reduced response burden through a reduced number of items [11]. We found the CAT did operate as a brief instrument for most participants, terminating for the majority (81.7%) by seven items, with an overall mean of 6.4 items answered. However, we allowed a minimum of one item (maintaining standard pre-specified precision levels) to enable the CAT to use as few items as possible, whereas the standard protocol allows a minimum of four items. The overall response burden of the CAT was not substantially better than the GAD-7 or HADS-A, which also outperformed the CAT on diagnostic statistics.

Respondents with an anxiety disorder completed fewer items on the PROMIS-A-CAT, as we found previously for patients with depression on the PROMIS-D-CAT [23]. This is consistent with CAT discontinuation rules, and has the practical effect of requiring fewer items to confirm the presence of more severe symptoms, and needing more items in the presence of less severe symptoms.

### Strengths and limitations

Strengths include comparison with legacy measures along with structured clinical interview and blinding of clinical interviewers to responses on the self-report measures. Additionally, only one other located study reported using the PROMIS-CAT software in oncology. Most studies using PROMIS measures in oncology have used SF measures. Although the sample size was reasonable, as was the number of participants with “Any Anxiety Disorder”, there is considerable heterogeneity in symptoms across anxiety disorders and further study of validity in specific sub-types of anxiety is warranted. Some types of anxiety disorders (social phobia, obsessive–compulsive disorder) may affect a person’s response to having cancer, but may not be the intended target of psycho-social programs attempting to address cancer-related anxiety. The study involved a time commitment from participants of one to two hours, which may have contributed to the relatively low consent rate (41%), so the sample may not be representative of all oncology patients in our clinic. However, to bias our results and conclusions, any resultant sample selection would have to affect the relationship between responses to the diagnostic interview and the self-report survey questions. Presenting the measures in a random order would have avoided potential order effects; as discussed previously [23], we believe there was limited likelihood of order effects. Completion rates for the measures were uniformly high (98–100%), and were not lower for measures presented later in the assessment, indicating there was no order-induced respondent fatigue. Formal measures of inter-rater reliability were not obtained; however, the SCID interview was administered by experienced psychologists, trained according to SCID protocol and the structured nature of the interview reduces variation.

## Conclusion

This study demonstrated the criterion validity of the PROMIS-A-CAT and PROMIS-A-SF measures relative to structured clinical interview (SCID) and convergent validity relative to several legacy measures in an oncology sample. The PROMIS measures had convergent and criterion validity similar to, but no better than, two well-established legacy measures of anxiety (GAD-7 and HADS-A), and somewhat better than the other legacy measures (DASS-A, DASS-S, DT, PSYCH-6). More work is needed to determine optimum cut-points on PROMIS anxiety measures and whether the T-score algorithm, based on US population norms, is optimal for oncology samples. Contrary to expectations, the PROMIS-A-SF demonstrated slight advantages over the PROMIS-A-CAT in terms of diagnostic accuracy and response burden.

## Reporting guidelines

The manuscript conforms to the guideline for reporting Diagnostic Accuracy Studies: STARD.

## Data Availability

The datasets used and/or analysed during the current study are available from the corresponding author on reasonable request.
